# A case of brucellosis-induced Guillain–Barre syndrome

**DOI:** 10.1186/s12879-021-07025-3

**Published:** 2022-01-20

**Authors:** Qian Li, Jianfeng Liu, Wenhui Jiang, Lisheng Jiang, Mengzhi Lu, Linping Xiao, Yukun Li, Yinghua Lan, Yongguo Li

**Affiliations:** 1 Present Address: Department of Infectious Diseases, The First Affiliated Hospital of Harbin Medical University, Harbin, China; 2 Present Address: Department of Neurology, The First Affiliated Hospital of Harbin Medical University, Harbin, China; 3 Department of Infectious Diseases, The Second Affiliated Hospital of Chongqing Medical University, Chongqing, China; 4 Present Address: Department of Infectious Diseases, The First Affiliated Hospital of Chongqing Medical University, Chongqing, China

**Keywords:** Brucellosis, Guillain–Barré syndrome, Diagnosis and treatment, Case Report

## Abstract

**Background:**

Guillain–Barre syndrome (GBS) is a rare neurological complication of brucellosis, and neurobrucellosis is the most common, but they have many similarities in clinical manifestations. Many clinicians are accustomed to merely explaining the manifestations of nervous system involvement with neurobrucellosis, but they ignore the possibility of GBS, and this leads to misdiagnosis, untimely treatment, and serious consequences.

**Case presentation:**

A 55-year-old male patient was admitted to The First Affiliated Hospital of Harbin Medical University for intermittent fever, fatigue, and waist pain more than three months. Brucellosis was diagnosed from the blood test. Although anti-brucella treatment was given at the time of diagnosis, the disease continued to progress. At the time of the cerebrospinal fluid systematic physical examination and the neuroelectrophysiological test, acute motor sensory axonal neuropathy was diagnosed. The patient was given immediately administered immunoglobulin therapy. After three months of systemic treatment, the patient's muscle strength of the distal limbs gradually recovered. The numbness of the limbs eased slowly, and urination function and respiratory function returned to normal. He could sit by himself.

**Conclusions:**

The possibility of GBS should be closely monitored for when a brucellosis patient shows typical clinical manifestations of progressive muscle weakness, protein-cell separation of the cerebral spinal fluid, and typical demyelinating sensorimotor polyneuropathy.

## Background

Neurologic changes have been detected in 3–5% of patients with brucellosis [1, 2]. The most common clinical manifestations of neurobrucellosis are fever, headache, and muscle rigidity. In addition, mental and motor sensory disorders may also occur. The culture of brucellosis and the detection of specific antibodies in the cerebrospinal fluid is the direct or indirect evidence for the diagnosis of neurobrucellosis [3, 4]. Guillain–Barre syndrome (GBS) is an immune-mediated peripheral neuropathy that is often induced by viruses and *Campylobacter jejuni* [5]. The primary lesion is a segmental demyelination of motor and sensory nerves in multiple nerve roots and peripheral nerves, however, brucella-induced GBS is very rare. The diagnosis and identification of the two complications caused by brucellosis presents a great challenge to clinicians.

## Case presentation

A 55-year-old male patient was admitted to the pain department of the First Affiliated Hospital of Harbin Medical University for intermittent fever. The maximum fever of 39.0 °C was accompanied by fatigue and waist pain for more than three months. He reported no nausea or vomiting.

Before he came to the First Affiliated Hospital of Harbin Medical University, the patient did not receive any drug but antipyretic analgesics to control the fever.

The patient raised sheep at his home; there had been an abortion and stillbirth of a lamb three months ago. The personal and family history were unremarkable. There was no history of upper respiratory tract infection or diarrhea.

The patient had long-term fever, fatigue, hyperhidrosis, and muscle and joint pain more than three months. He was admitted to the pain department. The doctor performed blood culture indicated an infection with gram-negative bacilli, and the tube agglutination test (SAT) titer was 1:400. He was diagnosed with brucellosis and given anti-brucella treatment. Four days later, the patient's fever gradually eased, but numbness and weakness began to appear on his legs. Symmetrical muscle weakness gradually appeared in the distal extremities, and this patient gradually became unable to move. He was then transferred to the department of infectious diseases.

Once the patient was admitted to our department, cerebrovascular disease, GBS, and neurobrucellosis were highly suspected. We performed a system physical examination including a neurological examination that showed mild paresthesia and muscle weakness. The muscle strength level of the arms was 0, the proximate muscle strength level of the legs was 0, and the distal muscle strength level was 2. A neuroelectrophysiological examination suggested that the limb F wave latency was extended and the nerve sensory conduction velocity of the legs was slowed down. Meningeal irritation signs and pyramidal signs were all negative.

An imaging examination, including magnetic resonance imaging (MRI), showed a degenerative cervical change. There were no infarction or hemorrhage observed in a computed tomography (CT) scan of the brain.

A laboratory examination was performed once the patient was admitted to our hospital. It showed mild liver damage and mild inflammatory activity: 2018-0-18 ALT:33.5 U/L, AST:41.7 U/L, ALB:33.3 g/L, GGT:63.7 U/L, WBC:7.92 × 109/L NEUT:7.01 × 109/L, NEUT%:88.4, MONO%:8, MONO:0.63 × 109/L, EO:0.00 × 109/L, EO%:0.00, CRP: 6.92 mg/L, ESR: PCT:0.11 ng/mL. The brucellosis agglutination test was retested, the titer of which was 1:1600. The IgM antibodies titer of rubella virus, cytomegalovirus, and herpes simplex virus were all negative.

Although anti-brucellosis drugs were given immediately, this patient's symptoms were not effectively controlled. Slight sensory disturbances, such as numbness and burning sensations, occurred at the end of the extremities. Muscle tone and tendon reflexes were absent in all limbs. Clinical manifestations of cranial nerve injury and autonomic nerve dysfunction included dysuria, sweating, dysphagia, ventilator paralysis, and neck weakness also gradually emerged.

**Fig. 1:**
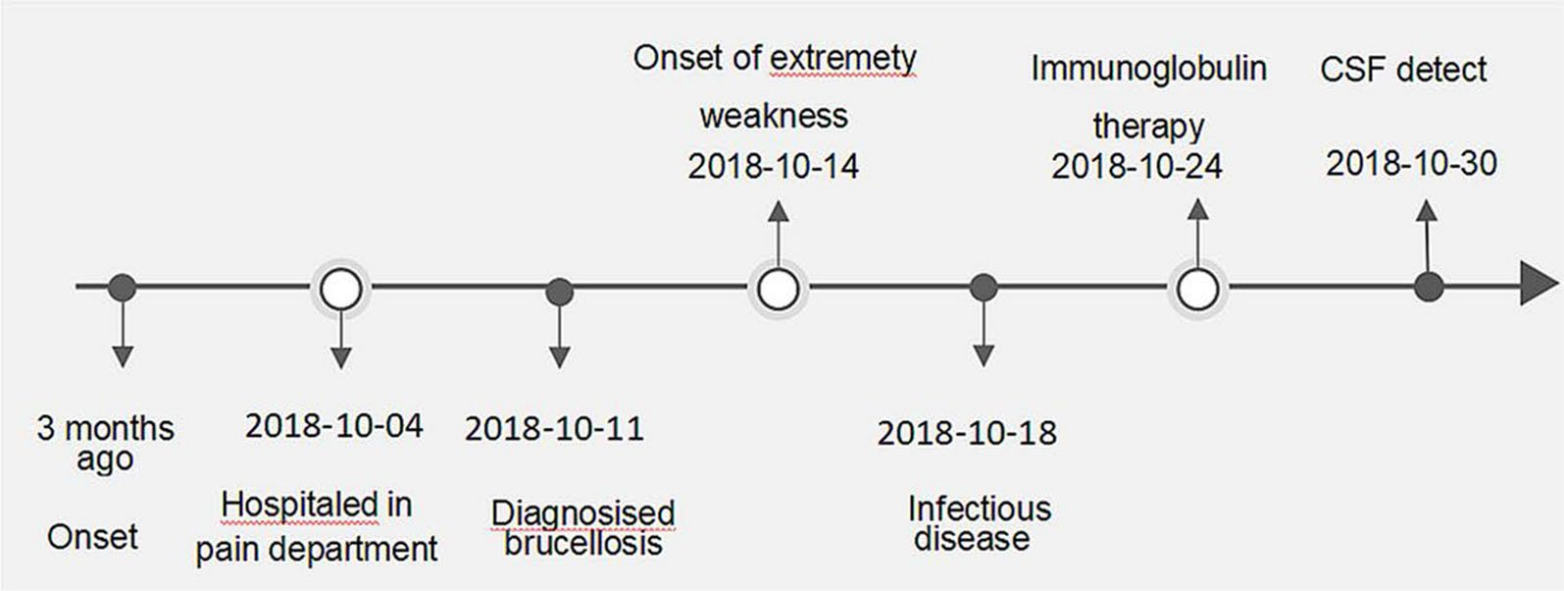
The timeline of the patient's diagnosis and treatment process

Due to the high suspect of GBS and that the disease was progressing rapidly, intravenous immunoglobulin therapy (25 g/day for six days) combined with neurotrophic nutrition were applied to patient on the 6th day after he was transferred to the department of infectious diseases. On the 12th day, we performed a lumbar puncture and some related CSF examinations on the patient (Fig. 1). In addition, the anti-GM1 antibody IgG, IgM and anti-GD1b antibody IgG, IgM of blood, and CSF were all positive. Additionally, the SAT and bacterial culture of the CSF were also negative. These findings further confirmed the diagnosis of AMSAN and excluded other possibilities that can lead to neuropathy (Table 1).

**Table 1:** Cerebrospinal fluid examinations

Cerebrospinal fluid		Reference range
Biochemical examination		
Total protein (mg/L)	2273.1	150.00–450.00
Chlorine (mmol/L)	118	119.00–129.00
Glucose (mmol/L)	4.64	2.34–3.66
Routine		
Total protein quantitation (mg/dL)	> 300.00	12.00–60.00
Glucose quantification (mg/dL)	78	40.00–70.00
Total number of cells (× 106)	10	0.00–8.00
Pathogen examinations		
Mycobacterium tuberculosis DNA assay	Not detected	
Ink staining	No cryptococcus	

With a diagnosis of brucellosis, the patient was given a course of antibiotic therapy (streptomycin (1 g/d i.m. 2w) + doxycycline (200 mg/d p.o. 12w) + trimethoprim-sulfa-ethoxazole (1.9 2 g/d p.o. 12w) + ceftriaxone sodium (2 g/d ivgtt 2w)). For the treatment of GBS, in addition to intravenous immunoglobulin and neurotrophic therapy, the patient was also given active rehabilitation (including muscle strength enhancement training, range of motion training, and acupuncture), and appropriate psychological counseling.

The patient's wife thought that the treatment of the patient was relatively timely, the patient's condition was not further aggravated, and the symptoms of urinary incontinence were gradually alleviated. Ten days later, the patient could take liquid food without choking and could perform some slight movements of the fingertips. After three months of systemic treatment, the patient's muscle strength of the distal limbs gradually recovered. The numbness of the limbs eased slowly, and urination function and respiratory function returned to normal. He was able to sit alone without assistance.

## Discussion and conclusion

Brucellosis is a zoonotic disease that affects the central and peripheral nervous systems with different neurological manifestations. Central nervous system lesions are more common, often acute, and present as meningoencephalitis. Peripheral nervous system lesions may be acute or chronic, often presenting as multiple radiculopathy. Currently, some scholars believe that the latter may be related to infection-induced immune system-mediated demyelination. GM1 ganglioside epitopes have been found on the surface of brucella, and anti-GM1 ganglioside antibodies have been detected in serum of mice immunized with *Brucella melitensis* [6]. Therefore, based on the molecular mimicism hypothesis, it was found that *Brucella* infection was a precipitator of GBS, which is biologically reasonable because ganglioside exists in the nerve membrane [7]. However, due to the small number of related cases, the pathogenesis requires further research [8, 9]. A few cases of GBS associated with brucellosis have been reported in the literature in the past. A 26-year-old woman and a 54-year-old man reported a GBS-related *Brucella* infection with upper and lower limb numbness, muscle weakness, and eye and facial weakness associated with GBS. Both achieved satisfactory results after appropriate immunoglobulin therapy [10, 11]. Alanazi et al. reported 19 patients with GBS during active brucellosis in 2021. Six of them were consistent with the axon form. Another eight patients were consistent with the demyelination pattern, three patients (15.8%) received intravenous immunoglobulin (IVIG) alone, seven patients (36.8%) received plasma exchange (PLEX) alone, three patients (15.8%) received both IVIG and PLEX, and six patients (31.6%) received neither IVIG nor PLEX. All of the patients were treated with antibiotics. One patient (5.3%) died, and 16 patients (84.2%) recovered their walking ability. Walking recovery time varied from two weeks to one year [12].

The diagnosis of GBS primarily depends on the typical clinical manifestations, typical protein-cell separation of the CSF, and neuroelectrophysiological changes [13]. The presence of anti-ganglioside antibodies is supportive evidence [14, 15]. There was no obvious abnormality in the entire spine MRI and the head CT scan of this patient. The results of CSF showed a typical protein-cell separation changes, but the pathogen related examinations of CSFwere all negative (Table 1). In addition, the anti-GM1 antibody IgG, IgM and anti-GD1b antibody IgG, IgM of blood, and CSF were all positive. The neuroelectrophysiological showed that the limb F wave latency was extended. This all supports diagnosis of Acute motor sensory axonal neuropathy (AMSAN) secondary to acute brucellosis.

In principle, implement immunotherapy as early as possible after the onset of GBS can help control the progression of the disease and reduce disability. Previous international research evidence on IVIG and plasma exchange therapy for GBS have primarily originated from typical GBS patients who were unable to walk independently (or whose condition was more severe) within two weeks of onset, suggesting that immunotherapy should be initiated as soon as possible [16]. However, considering the relevant guidelines and recommendations, the time for the occurrence of specific antibodies and typical protein-cell separation changes in the CSF of GBS patients should be two to four weeks after the onset [17]. Considering that the optimal time for treatment of such patients is earlier than that of typical CSF changes, immun- -oglobulin intramuscular treatment was implemented prior to performing the lumbar puncture, and this would have controlled further deterioration from the disease.

Both immunoglobulin and plasma exchange are the first choices for the treatment of GBS, and if the first round of plasma exchange in patients with severe GBS there is a poor effect after the end of the immunoglobulin therapy, doctors can try a second round of continued treatment. However, there is not enough evidence to prove that the combination therapy is better. Hence, plasmapheresis was not performed in this patient [18].

Fatigue occurs in most patients with GBS and is not related to disease severity and can last for several years. Studies have found that physical therapy and rehabilitation exercises, such as cycling, are effective for reducing symptoms. Recent studies have demonstrated that acupuncture can counteract skeletal muscle atrophy by increasing IGF-1 levels and by stimulating muscle regeneration [19, 20]. A 2021 study by Shou et al. demonstrated that a therapeutic regimen of acupuncture combined with rehabilitation training is more effective than the rehabilitation training alone for the treatment of ICU-acquired weakness (ICUAW) [21]. Because relevant studies have primarily been limited to China and the sample sizes were small, the exact curative effect of acupuncture treatment requires further confirmation. Therefore, it is not recommended for regular use but is only recommended as an alternative.

The case fatality rate of GBS is as high as 4–15%, and nearly 20% suffer from persistent disability. These disability cases can be divided into several subtypes, among which AMSAN is primarily characterized by motor and sensory axonal degeneration of the nerve roots and peripheral nerves, and the clinical manifestations are typically the most severe [22–24]. The patient was eventually diagnosed as AMSAN. His condition was more severe and and required a longer recovery time than other similar cases have reported previously. It could deduce that there existed mild limb weakness and fatigue in the early stage of GBS, but the physicians misidentified them as the fatigue syndrome associated with brucellosis. Would patient outcomes be better if they could be treated with immunoglobulin earlier? In the department of infectious diseases, there is often a lack of comprehensive understanding of the abnormal signs of the nervous system, which to some extent is responsible for the delay in diagnosis. This is probably an inevitable flaw in our diagnosis and treatment process. It is necessary to pay more attention to such patients in clinic in order to raise the rate of early diagnosis.

In conclusion, the possibility of GBS should be monitored for closely when a brucellosis patient displays typical clinical manifestations of ascending muscle weakness, protein-cell separation of the CSF, and typical demyelinating sensorimotor polyneuropathy.

## Data Availability

The dataset supporting the conclusions of this article is included in the article.

## Abbreviations

GBS: Guillain–Barre syndrome
SAT: Tube agglutination test
MRI: Magnetic resonance imaging
CT: Computed tomography
CSF: Cerebrospinal fluid
AMSAN: Acute motor sensory axonal neuropathy
ICUAW: ICU-acquired weaknes
IVIG: Intravenous immunoglobulin
PLEX: Plasma exchange
